# Testicular shear wave elastography in varicocele: measurement strategy and clinical application

**DOI:** 10.1186/s12610-026-00310-8

**Published:** 2026-04-01

**Authors:** Lisu Zhang, Yi Li, Lingyan Zhang, Yinying Liang, Yumin Zhuo, Jun Huang

**Affiliations:** 1 Department of Ultrasound, The Second Affiliated Hospital of Guangzhou University of Chinese Medicine (Guangdong Provincial Hospital of Chinese Medicine), Guangzhou, China; 2 Department of Ultrasound, The Affiliated Guangdong Second Provincial General Hospital of Jinan University, Guangzhou, China; 3 Department of Ultrasound, The First Affiliated Hospital of Jinan University, Guangzhou, China; 4 Department of Urology, The First Affiliated Hospital of Jinan University, Guangzhou, China

**Keywords:** Varicocele, Shearwave elastography, Influencing factors, ROI, Probe, Ultrasound, Varicocèle, Elastographie par Ondes de Cisaillement, Facteurs d’Influence, Région d’Intérêt, Sonde, Echographie

## Abstract

**Background:**

Conflicting results on testicular stiffness in varicocele and the lack of a standardized measurement protocol hinder clinical staging. In this study, we aimed to investigate the effects of probe frequency and region of interest (ROI) size selection on testicular stiffness measurements and to evaluate the testicular parenchymal stiffness and volume changes in patients with varicocele compare with healthy controls under standardized conditions.

**Results:**

Shear wave elastography (SWE) was first performed on 62 testes from 31 healthy men, with two probe frequencies (SL10–2 and SL15–4) and four ROI sizes (4, 6, 8, and 10 mm), to assess the impact on SWE measurements. Based on the lowest variability settings, testicular volume and stiffness were compared in 76 patients with varicoceles and 68 healthy controls. Different SWE values were obtained with the two probes and four ROI sizes (*p* < 0.05). The SL15–4 probe showed lower variability, with the lowest coefficient of variation at the 4 mm ROI. The median testicular SWE value was 2.03 (1.97–2.07) kPa in the control group and 2.01 (1.87–2.07), 1.77 (1.53–1.98), and 1.51 (1.45–1.63) kPa in the Grade I, II, and III varicocele groups, respectively. SWE values were significantly lower in varicocele patients and decreased with increasing grade. The median testicular volumes were 13.08 (12.06–15.70) mL in the control group and 12.26 (10.70–15.15), 13.06 (11.34–14.72), and 11.84 (11.26–12.86) mL in the Grade I, II, and III varicocele groups, respectively. No significant difference in volume was observed between varicocele and control groups (*p* = 0.387).

**Conclusion:**

Probe frequency and ROI size influence testicular SWE measurements, and caution should be exercised when comparing SWE results. Our study showed that testicular stiffness was decreased in patients with varicocele without significant changes in testicular volume, suggesting the presence of subtle structural alterations. SWE may provide complementary information by detecting changes in testicular parenchymal in patients with varicocele.

## Résumé

**Contexte:**

Des résultats contradictoires sur la raideur testiculaire en présence de varicocèle ainsi que l’absence d’un protocole de mesure standardisé freinent la stadification clinique. Dans la présente étude, nous avons cherché à étudier les effets de la fréquence de la sonde et de la sélection de la taille de la région d’intérêt (ROI) sur les mesures de raideur testiculaire, ainsi qu’à évaluer la raideur et les variations de volume du parenchyme testiculaire chez les patients porteurs de varicocèle par rapport à des témoins sains, dans des conditions standardisées.

**Résultats:**

L’élastographie par ondes de cisaillement (SWE) a d’abord été réalisée sur 62 testicules chez 31 hommes en bonne santé, avec deux fréquences de sonde (SL10–2 et SL15–4) et quatre tailles de la ROI (4, 6, 8 et 10 mm), afin d’évaluer l’impact sur les mesures par SWE. Sur la base des réglages de variabilité les plus faibles, le volume et la raideur testiculaires ont été comparés chez 76 patients atteints de varicocèles et 68 témoins sains. Différentes valeurs de SWE ont été obtenues avec les deux sondes et quatre tailles de ROI (p < 0,05). La sonde SL15–4 a montré une variabilité plus faible, avec le coefficient de variation le plus faible pour une ROI de 4 mm. La valeur médiane de SWE testiculaire était de 2,03 (1,97–2,07) kPa dans le groupe témoin et respectivement de 2,01 (1,87–2,07), 1,77 (1,53–1,98) et 1,51 (1,45–1,63) kPa dans les groupes de varicocèles de grade I, II et III. Les valeurs de SWE étaient significativement plus faibles chez les patients avec varicocèles et diminuaient avec l’augmentation du grade. Les médianes des volumes testiculaires étaient de 13,08 (12,06–15,70) mL dans le groupe témoin et respectivement de 12,26 (10,70–15,15), 13,06 (11,34–14,72) et 11,84 (11,26–12,86) mL dans les groupes de varicocèles de grade I, II et III. Aucune différence significative de volume testiculaire n’a été observée entre les groupes varicocèle et témoins (*p* = 0,387).

**Conclusion:**

La fréquence de la sonde et la taille de la ROI influencent les mesures de la SWE testiculaire, et la prudence est recommandée lors de la comparaison des résultats de la SWE. Notre étude a montré que la raideur testiculaire était diminuée chez les patients porteurs de varicocèle sans changement significatif du volume testiculaire, suggérant la présence de subtiles altérations structurelles. La SWE peut fournir des informations complémentaires en détectant des modifications du parenchyme testiculaire chez les patients avec varicocèle.

## Introduction

Varicocele (VC) is a common vascular abnormality of the pampiniform plexus, affecting 15%–20% of men in the general population and up to 80% of those with secondary infertility [1]. Prolonged varicocele may increase testicular temperature, oxidative stress, and toxic metabolite reflux, leading to testicular atrophy, impaired spermatogenesis, and reduced fertility [2].

Male infertility associated with varicocele is potentially reversible. Surgical ligation and embolization are considered effective, particularly in men with abnormal semen parameters or testicular atrophy. Their impact on fertility remains controversial [3], as not all patients benefit from surgery. Varicocele may exert progressive detrimental effects on the testis [4]. Routine follow–up for asymptomatic patients generally includes semen analysis and ultrasound assessment of testicular volume [5]. However, semen analysis may be subject to considerable variability [6], and testicular atrophy has been suggested to develop gradually during disease progression [7], which limits the early detection of parenchymal alterations.

Color doppler flow imaging (CDFI) is the preferred imaging modality for the diagnosis and postoperative follow–up of varicocele [8]. However, it only reflects venous reflux and intratesticular hemodynamic changes, without information on structural or pathological alterations. Testicular biopsy is the gold standard but is invasive and unsuitable for routine use [9]. Therefore, a reliable noninvasive imaging technique may be useful for evaluating testicular parenchymal changes associated with varicocele and assist urologists in identifying patients at risk of infertility.

Shear wave elastography (SWE) is a noninvasive ultrasound technique that measures tissue stiffness by tracking shear wave propagation and reconstructing real–time elastograms. Stiffer tissue conducts shear waves faster [10]. SWE has been applied in liver, thyroid, and breast diseases [11, 12], and growing evidence supports its use in testicular lesion. Higher SWE values have been linked to orchitis, malignancy, and torsion, indicating potential for distinguishing normal from abnormal testes [13]. Recent studies suggest SWE may also predict fertility in patients with varicocele. Some found a negative correlation between stiffness, testicular volume, and semen quality [14, 15], while others reported decreased stiffness without significant volume changes or correlations with semen parameters [16, 17].These inconsistencies may reflect differences in patient cohorts, disease stages, or measurement methods.

Research on testicular SWE in relation to varicocele remains limited. More importantly, the absence of a consensus protocol for measuring testicular stiffness has resulted in considerable variability in the reported values for both normal and diseased testes. These inconsistencies not only hinder the clinical interpretation of SWE findings by urologists but also complicate the comparison of outcomes in long–term follow–up.

Therefore, this study is the first to evaluate the effects of probe frequency and ROI size on testicular SWE measurements, providing methodological insights into factors that may influence measurement variability. In addition, we compared testicular stiffness and volume between patients with varicocele and healthy controls to explore the clinical value of SWE in diagnosis and management.

## Materials and methods

### Research subjects

The local institutional review committee reviewed and approved the study protocol. Written informed consent was obtained from all the participants.

This prospective comparative study was conducted between October 2024 and January 2025 and included 175 men, including patients with left sided varicocele and healthy individuals. All participants underwent clinical physical examination performed by a urologist and scrotal ultrasound assessment conducted by an experienced ultrasonographer on the same day. The exclusion criteria were as follows: (1) presence of right–sided or bilateral varicocele; (2) testicular conditions such as microlithiasis, cysts, orchitis, tumours, torsion, or hydrocele of the testicular sheath; (3) cardiovascular or cerebrovascular diseases that rendered the participant unable to cooperate with the examination. The control group included individuals with normal findings on both physical examinations and scrotal ultrasound. The same exclusion criteria were also applied to the control group.

### Ultrasound examination methods and contents

SWE was performed using an ultrasound diagnostic imaging instrument, Aixplorer (Super Sonic Imagine, Aix–en–Provence, France), equipped with SL10–2 MHz and SL15–4 MHz linear probes. All measurements were performed by a single ultrasonographer with over five years of specialized experience in SWE.

All measurements were performed with the patients in the supine position. Conventional grayscale ultrasound was first conducted in transverse and longitudinal planes to evaluate the testicular parenchyma and assess exclusion criteria. Testicular volume was subsequently measured by obtaining the length and height on the maximal longitudinal section and the width on the maximal transverse section. The testicular volume was measured using Lambert's formula (length × width × height × 0.71) [18].

Based on our clinical experience, varicocele was diagnosed using the Chiou system [19], which evaluates maximum vein diameter, pampiniform plexus characteristics, and changes in venous flow during the Valsalva maneuver. A cumulative score ≥ 4 was considered indicative of varicocele (Table 1). The severity of varicocele was classified according to the method specified by Dubin and Amelar [20]: Grade I, veins palpable only during the Valsalva manoeuvre; Grade II, varicose veins palpable while breathing normally; and Grade III, varicose veins noticeable on visual examination at rest. Physical examination was used to determine the clinical varicocele grade, whereas ultrasound assessment based on the Chiou scoring system was applied to confirm the presence of varicocele in cases of discrepancy, particularly for Grade I varicocele.

**Table 1:** Chiou scoring system for ultrasound diagnosis of varicocele

Parameter	Criteria	Score
Maximum vein diameter (mm)	< 2.5	0
	2.5–2.9	1
	3.0–3.9	2
	≥ 4.0	3
Plexus/sum of diameter of veins	No plexus identified	0
	Plexus identified with sum diameter < 3 mm	1
	Plexus identified with sum diameter 3–5.9 mm	2
	Plexus identified with sum diameter ≥ 6 mm	3
Change of flow velocity on Valsalva maneuver	< 2 cm/s or duration < 1 s	0
	2–4.9 cm/s	1
	5–9.9 cm/s	2
	≥ 10 cm/s	3

**Fig. 1:**
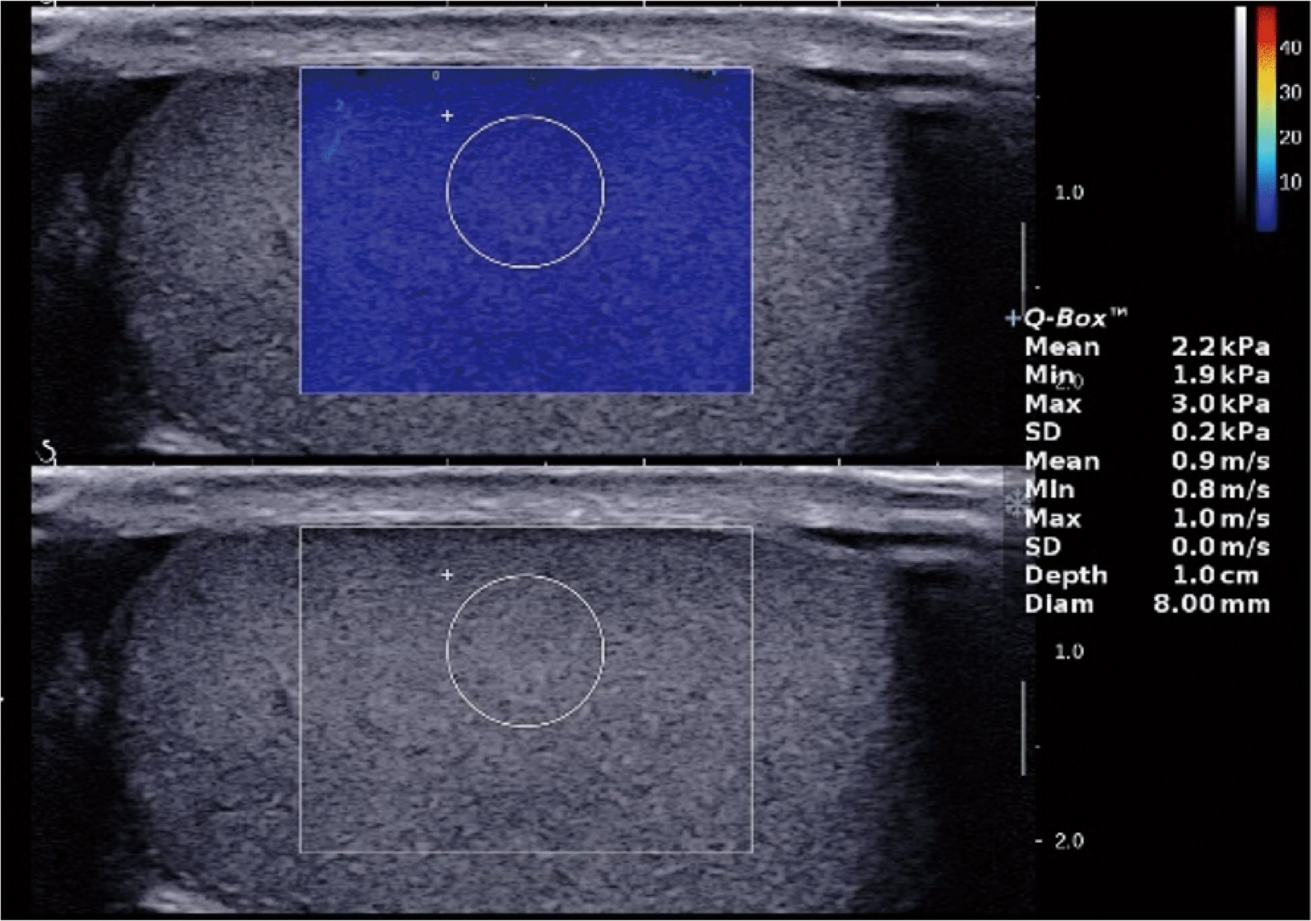
Shear wave elastography image placed in the longitudinal plane of the testis. The ROI was placed at 1 cm in the central parenchyma, and the mean stiffness value (Emean, kPa) was recorded. Measurements were repeated three times to assess testicular stiffness, and the average value was used

**Fig. 2:**
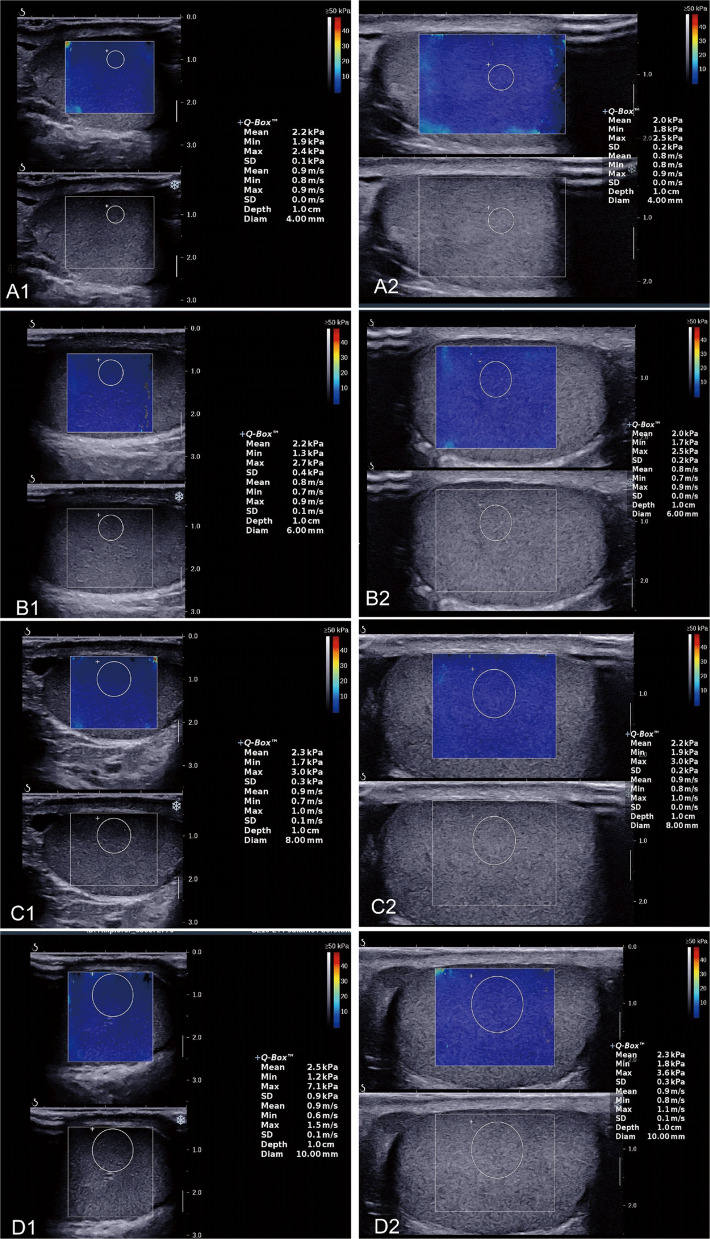
Shear wave elastography image of the testicle in a 27–year–old healthy man showing SWE values obtained using two linear probes at different frequencies and four ROI diameters. A1–D1 indicate SWE values measured with the SL10–2 probe at ROI diameters of 4, 6, 8, and 10 mm. A2–D2 indicate SWE values measured with the SL15–4 probe at the same ROI diameters

Finally, all enrolled participants underwent SWE examination. Adequate coupling gel was applied, and minimal transducer pressure was maintained. ROI placement was standardized at 1 cm within the central testicular parenchyma on the maximal longitudinal section, avoiding the mediastinum testis and tunica albuginea (Fig. 1). SWE measurements were performed on 62 testes (from 31 healthy men) using two probes (SL10–2 and SL15–4) with four regions of interest (ROI) sizes (4, 6, 8, and 10 mm). The mean elasticity value (Emean) was recorded (Fig. 2). Three consecutive measurements were taken and the average of the three measurements was used for subsequent analysis. The coefficient of variation (CV) was calculated for each group. Finally, testicular stiffness in the varicocele and control groups was obtained by combining the probe and ROI with the minimum CV.

### Statistical analysis

Statistical analysis was performed using SPSS version 25.0 (IBM Corp., Armonk, NY, USA). Data are presented as mean ± standard deviation (M ± SD) and median (IQR). Normality of data distribution was assessed using the Kolmogorov–Smirnov test, and the homogeneity of variances was evaluated using Levene’s test. For variables with a normal distribution, comparisons among multiple groups were performed using one way analysis of variance (ANOVA), followed by Bonferroni post hoc tests. Student’s t–test was applied to evaluate the effect of different probe frequencies on SWE values at the same ROI size. For variables that did not follow a normal distribution, the Kruskal–Wallis test was used to evaluate overall differences among groups, and pairwise group comparisons were performed using Mann–Whitney U test. Benjamini–Hochberg method is used to correct the statistical significance of multiple comparisons. Measurement variability was assessed using the coefficient of variation (CV), with lower CV values indicating lower variability of the SWE measurements. Correlations between testicular SWE values and volume were assessed using Spearman rank correlation coefficients analysis. Statistical significance was set at *P* < 0.05.

## Results

A total of 175 participants were included in the study, comprising 99 healthy men and 76 patients with left sided varicocele (Grade I, n = 29; Grade II, n = 22; Grade III, n = 25). A total of 206 testes were evaluated. For the analysis of the effects of different probe frequencies and ROI sizes on SWE values in normal testes, 62 testes from 31 healthy men were included. For the evaluation of varicocele related changes in testicular stiffness and volume, only the left testes were analyzed, including 76 testes from the varicocele group and 68 testes from the control group.

The mean age was 29.5 ± 5.20 years in the control group, 29.1 ± 9.80 years in the Grade I varicocele group, 28.7 ± 5.01 years in the Grade II varicocele group, and 28.7 ± 9.00 years in the Grade III varicocele group. There was no significant difference in age between the varicocele and control groups (*p* = 0.450, Table 2).

**Table 2:** Comparison of age between the control and varicocele groups

Group	Control group (*n* = 68)	VCI (*n* = 29)	VCII (*n* = 22)	VCIII(*n* = 25)	*P* value
Year(M ± SD)	29.5 ± 5.20	29.1 ± 9.80	28.7 ± 5.01	28.7 ± 9.00	0.450

Analysis of variance (ANOVA) showed no significant difference in the mean age between the control and varicocele groups. *VC* varicocele, VCI, VCII, and VCIII represent the left grades I, II, and III varicocele, respectively; M ± SD, mean ± standard deviation

### SWE values varied significantly by probe and ROI size

Measurements obtained with the SL10–2 probe were higher than those from the SL15–4 probe (*p* < 0.001; Table 3). SWE values increased with ROI size for both probes, though the difference between 4 and 6 mm ROIs was not significant (p = 0.313; p = 0.260 Tables 3 and 4). The SL15–4 probe showed lower variability, with the lowest coefficient of variation observed at 4 mm ROI.

**Table 3:** Testicular SWE values and CV in two probes and four ROI sizes

ROI (mm)	SWE (Kpa)		P value	CV (%)	
	SL10–2	SL15–4		SL10–2	SL15–4
4	2.15 ± 0.103	2.00 ± 0.090	< 0.001	4.7	4.5
6	2.20 ± 0.120	2.04 ± 0.095	< 0.001	5.4	4.6
8	2.27 ± 0.127	2.15 ± 0.100	< 0.001	5.5	4.6
10	2.43 ± 0.140	2.23 ± 0.110	< 0.001	5.8	4.9
P value	< 0.001	< 0.001			

The data are expressed as mean ± standard deviation. Analysis of variance (ANOVA) demonstrated statistically differences in the testicular SWE values obtained by using different ROI sizes. The t–test revealed statistically differences in testicular SWE values between the SL10–2 and SL15–4 probes within the same ROI size. *SWE* shear wave elastography, *ROI* region of interest, *CV* coefficient of variation

**Table 4:** Comparison of testicular SWE values with different ROIs and probes

ROI (mm)	P value	
	SL10–2	SL15–4
4 VS 6	0.313	0.260
4 VS 8	< 0.001	< 0.001
4 VS 10	< 0.001	< 0.001
6 VS 8	0.016	< 0.001
6 VS 10	< 0.001	< 0.001
8 VS 10	< 0.001	< 0.001

*SWE* shear wave elastography, *ROI* region of interest

VS represents the comparison of SWE values between ROI sizes in two probes

**Fig. 3:**
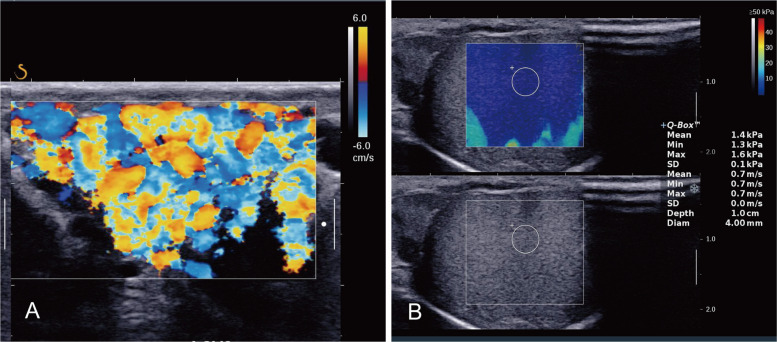
A 28–year–old man diagnosed with grade III left varicocele. A: Color doppler flow imaging of the left spermatic vein. B: SWE value of the left testis was 1.4 kPa, measured using the SL15–4 probe with a 4 mm ROI

To minimize measurement variability, SWE was performed on the left testis of 68 healthy controls and 76 patients with varicocele using the SL15–4 probe with a 4 mm ROI (Fig. 3).

The median testicular stiffness was 2.03(1.97–2.07) kPa in the control group, and 2.01(1.87–2.07) kPa in the Grade I varicocele group, 1.77(1.53–1.98) kPa in the Grade II varicocele group, and 1.51(1.45–1.63) kPa in the Grade III varicocele groups. Compared with the control group, patients with varicocele exhibited lower SWE values (*p* < 0.001). SWE values progressively decreased with increasing varicocele grade, showing a negative correlation with severity (r = –0.692, *p* < 0.05). In further pairwise comparisons between the control group and patients with different grades of varicocele (Grades I, II, and III), the SWE value in patients with Grade I varicocele was slightly lower than that in controls. However, the difference was not statistically significant (*p* = 0.170) (Table 5).

**Table 5:** Comparison of SWE values between control and varicocele groups

Group	Adjusted p value
Control group VS VCI	0.170
Control group VS VCII	< 0.006
Control group VS VCIII	< 0.003
VCI VS VCII	0.006
VCI VS VCIII	< 0.002
VCII VS VCIII	0.028

VS represents the comparison of testicular SWE values between different groups using the Mann–Whitney U test. Benjamini–Hochberg method is used to correct the statistical significance of multiple comparisons. *SWE* shear wave elastography, *VC* varicocele, *VCI,* VCII, and VCIII represent the left grades I, II, and III varicocele, respectively

Median testicular volumes were 13.08 (12.06–15.70) mL in controls group, and 12.26 (10.70–15.15), 13.06 (11.34–14.72), and 11.84 (11.26–12.86) mL in Grades I, II, and III varicocele group, respectively. No significant differences in volume were observed between the control and varicocele groups (*p* = 0.387; Tables 6).

**Table 6:** Comparison of SWE values and volumes between the control and varicocele groups

Group	Control group (*n* = 68)	VCI (*n* = 29)	VCII (*n* = 22)	VCIII (*n* = 25)	P value
SWE(Kpa)	2.03(1.97–2.07)	2.01(1.87–2.07)	1.77(1.53–1.98)	1.51(1.45–1.63)	< 0.001
Volume(mL)	13.08(12.06–15.70)	12.26(10.70–15.15)	13.06(11.34–14.72)	11.84(11.26–12.86)	0.387

The SWE values and volumes are presented as median (IQR). The Kruskal–Wallis H test revealed significant differences in testicular SWE values but no significant differences in testicular volume between the control and varicocele groups. *SWE* shear wave elastography, *VC* varicocele, VCI, VCII, and VCIII represent the left grades I, II, and III varicocele, respectively; Volume, left testicular volume

**Fig. 4:**
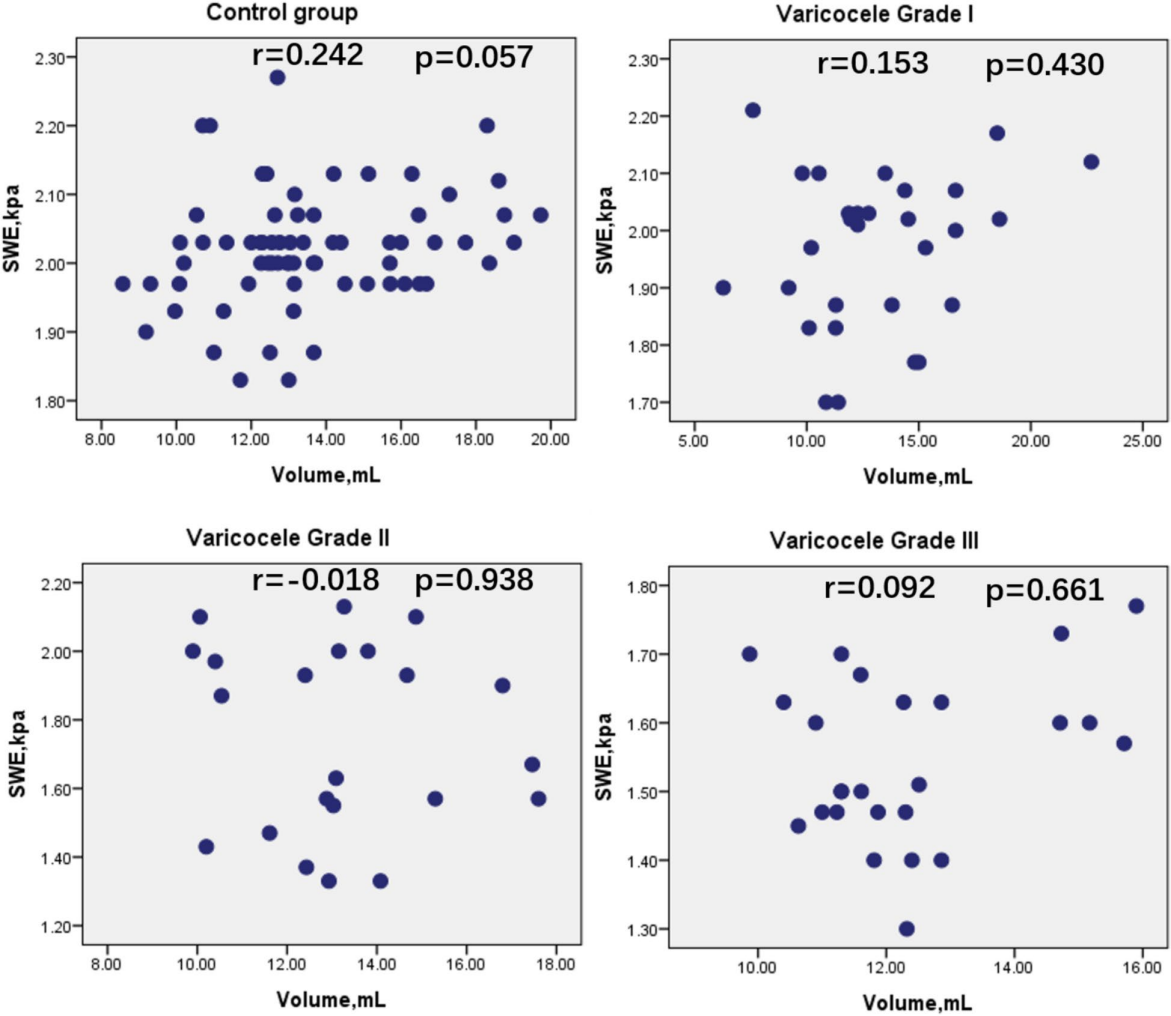
Correlation between testicular SWE value and volume in the control and varicocele groups

Pearson correlation analysis was performed to evaluate the correlation between testicular SWE values and volume in the control group and in each varicocele group. No significant correlation was found between SWE value and testicular volume in the control and varicocele groups (Control group: r = 0.242, p = 0.057; Grade I group: r = 0.153, p = 0.430; Grade II group: r = –0.018, p = 0.938; Grade III group: r = 0.092, p = 0.661) (Fig. 4).

Scatter plots show the correlation between SWE values and testicular volume in the control group and in patients with varicocele grades I, II, and III. The x-axis represents testicular volume (mL), and the y-axis represents SWE values (kPa). Correlation coefficients (r) and corresponding p values are shown in each panel. Pearson correlation analysis revealed no significant correlation between SWE values and volume in all groups. SWE, shear wave elastography; Volume, the testicular volume.

## Discussion

In this study, we investigated the effects of different probe frequencies and ROI sizes on testicular SWE values using the same machine. Our results demonstrated that testicular SWE values varied depending on probe frequency and ROI size. Testicular SWE values were higher when measured using the low frequency linear probe (SL10–2) compared with the high frequency probe (SL15–4), and SWE values increased with ROI size. For the Aixplorer system, measurements obtained using the SL15–4 linear probe with an ROI size of 4 mm showed lower measurement variability. We found that compared with healthy controls, patients with varicocele exhibited reduced testicular stiffness, whereas no significant difference in testicular volume was observed. These findings suggest that SWE can detect parenchymal alterations associated with varicocele before overt morphological changes in the testis.

Few previous studies on the application of SWE in the testes have analysed how the measurement method affects stiffness variability. In the first part of our study, we found that testicular SWE values obtained using the SL10–2 linear probe were higher than those measured with the SL15–4 probe. This finding is consistent with results from previous studies that investigated the effects of probe frequency on shear wave elastography measurements in phantoms and liver tissue. Dillman [21] evaluated the effects of different instruments and probes on shear wave velocity (SWV) in hard phantoms, and found that SWV values obtained using the SL10–2 probe was significantly higher than those measured with the SL15–4 probe. Additionally, in a study involving 96 pediatric patients, a significantly higher liver stiffness values were observed when measurements were performed with the low frequency probe (SC6–1) compared with the high frequency probe (SL15–4) [22]. The differences in the stiffness values may be attributed the vibration frequency, bandwidth, and spatial resolution of the probes [23]. Additionally, variations in the methods used to calculate the arrival time and velocity of the relative shear waves may also contribute to discrepancies in stiffness measurements [24]. Therefore, when comparing testicular stiffness values in clinical work, consistency in probe frequency should be taken into consideration.

Information on the influence of ROI size on testicular stiffness remains limited. In this study, we evaluated the effects of various ROI sizes (4, 6, 8, and 10 mm) on SWE measurements and the variability. Our results showed that the SWE value differ with different ROI sizes and increased with ROI size. Our findings support the conclusion of Rominger et al. [25] that ROI characteristics influence SWV measurements. Interestingly, in their study, increasing the ROI width resulted in a significant increase in muscle SWV values. This may be related to the use of a circular ROI in our study, as opposed to a rectangular ROI in the latter. Further analysis of our data indicated that testicular stiffness measured with 6 mm ROI was slightly higher than that obtained with 4 mm ROI, although the difference did not reach statistical significance. These results suggest that differences in reported testicular SWE values may be acceptable when the ROI size is between 4 and 6 mm. The reasons may be attributed to the following factors. The shear wave elasticity image is not entirely uniform. Typically, a softer blue region is observed in the centre, while higher stiffness is seen near the edges of the sampling box, appearing as green or yellow–green areas [24]. This occurs because the system can mistakenly display stiffer values at the edges of the region as the pulse is strongest at the centre and weaker at the sides, causing less tissue movement there. Furthermore, the fibrous capsule around the testes can cause variations in elasticity measurements [26]. Using a larger ROI may include these harder artefacts, leading to larger SWE values. Therefore, it is important to avoid these artefacts and choose a suitable ROI size. The CVs in our study were small, ranging from 4% to 6%, comparable to those reported by Chen et al. in a phantom study, in which CVs of 0%–7.16% were obtained with the SL15–4 and SL10–2 linear probes [27]. Our findings show that the SL15–4 probe combined with a 4–mm ROI was associated with lower measurement variability than the other acquisition settings.

In this study, there was no significant age difference between the control and varicocele groups, which excluded the influence of age on testicular stiffness. Some studies have demonstrated that testicular stiffness is positively related to age, suggesting that aging can cause thickening of the connective tissue sheath of the vas deferens as well as fibrosis between the vas deferens [28]. Therefore, age cannot be ignored while analysing and comparing testicular stiffness.

Currently, contradictory findings exist regarding changes in stiffness and volume in testes affected by varicocele. Several studies have reported that, compared with healthy testes, those in patients with varicocele exhibit increased stiffness and reduced volume [29–31]. In addition, investigations have described a significant increase in testicular stiffness when the testicular atrophy index (TAI) reaches more than 20% or in the presence of spermatogenic dysfunction [15, 32]. In contrast, other investigations have demonstrated lower testicular stiffness in patients with varicocele, without significant alterations in testicular volume [16, 33, 34]. Furthermore, some reports have observed no significant differences in either testicular stiffness or volume between varicocele and control groups [35, 36]. These inconsistent results may be attributed to multiple factors, including differences in sample size, elastography technique, ROI selection, and the stage of disease in patients with varicocele. Stiffness in certain studies was assessed using strain elastography [31], which relies on manual compression to induce tissue deformation. Variations in the applied pressure may result in differences in the measured stiffness values. In the study by Erdogan et al. [29], the ROI encompassed the entire testis, including the highly stiff tunica albuginea, which may have significantly influenced the stiffness measurements. Furthermore, the absolute stiffness values reported in these studies were higher than those observed in our study. On the other hand, the stage of varicocele progression may also be an important factor contributing to the variability in findings. In studies reporting increased stiffness, patients were often accompanied by testicular atrophy or impaired spermatogenic function. This may be attributed to chronic injury leading to fibrosis of the seminiferous tubule walls and atrophy of interstitial cells [37], thereby further compromising testicular function. Table 7 summarizes the previous literature on changes in testicular stiffness and volume in varicocele.

**Table 7:** The changes in testicular stiffness and volume reported in the literature on varicocele

Study	Elastography technique	Sample size (testes)	Testicular Stiffness in Varicocele	Testicular Volume in Varicocele
Erdogan et al	SWE	VC group(*n* = 50); contralateral normal testes group(*n* = 46); control group(*n* = 104)	Increased	Decreased
Li K et al	Strain elastography	VC I group(*n* = 66); VC II group(*n* = 60); VC III group(*n* = 70); control group(*n* = 50)	Increased	Decreased
Salbas et al	SWE	VC group(*n* = 78); contralateral normal testes group(*n* = 60); control group(*n* = 152)	Increased	Decreased
Turna et al	SWE	VC group(*n* = 116), VC group subdivided in two groups: normospermic and oligospermic group; control group(*n* = 58)	Increased; higher stiffness observed in oligospermic subgroup	Decreased
Jedrzejewski et al	SWE	VC group(*n* = 30); contralateral testes group(*n* = 30)	Increased only in cases with > 20% testicular volume reduction; no significant difference when volume reduction was 0%–20%	Decreased
Alperen et al	SWE	VC group(*n* = 55); control group(*n* = 69)	Decreased	No significant difference
Dede et al	ARFI	VC group(*n* = 30); control group(*n* = 30)	Decreased	No significant difference
Baleato–Gonzalez et al	SWE	VC group(*n* = 19); contralateral normal testes group(*n* = 13); control group(*n* = 44)	No significant difference	No significant difference
Yüzkan et al	SWE	VC group(*n* = 66); contralateral normal testes group(*n* = 50); control group(*n* = 116)	No significant difference	Decreased

*ARFI* Acoustic radiation force impulse, *SWE* Shear wave elastography

In our study, testicular stiffness was lower in patients with varicocele than in healthy controls and progressively decreased with increasing varicocele grade. However, no significant difference in testicular volume was observed between the two groups. Moreover, no significant correlation was found between testicular stiffness and volume, suggesting that alterations in stiffness may occur independently of changes in testicular volume. Our findings are consistent with those of Dede et al. [16] and Alperen et al. [34], who reported decreased testicular stiffness in patients with varicocele without significant differences in testicular volume. Interestingly, in our study, varicocele was stratified by severity (grades I, II, and III) and compared with the control group. We observed that testicular stiffness in grade I varicocele was slightly lower than that of controls, although the difference did not reach statistical significance. This may be because mild varicocele has not yet induced detectable changes in the testicular parenchyma measurable by SWE. These findings suggest that variations in testicular stiffness may reflect different stages of parenchymal alterations associated with varicocele. In the early stage, spermatic venous reflux may induce testicular swelling and decreased tissue density, which could account for the observed reduction in testicular stiffness. During the late stage, parenchymal cell injury and interstitial fibrosis may result in testicular atrophy, ultimately contributing to increased stiffness. These changes may be associated with the severity and duration of varicocele. The SWE values in our study, including both normal testes and affected testes were similar to those reported by Alperen et al. [34], despite differences in the ultrasound equipment used. Notably, the ROI size in their study was 3 mm, whereas ours was 4 mm, and in both studies, the ROI was placed in the midportion of the testis, avoiding the fibrous tunica, to obtain SWE values from a relatively homogeneous elastogram. Methodological differences such as these may partly account for discrepancies in stiffness values reported by other studies.

Surgery and percutaneous embolisation are the mainstay of treatment for varicocele, although their association with improved male fertility remains controversial [3]. Testicular volume atrophy and abnormal semen parameters are still considered surgical indications [5]. For young patients with clinical varicocele who have normal testicular volume and semen parameters but are concerned about future fertility, there is still uncertainty regarding the choice of surgical intervention or conservative treatment, as previous studies have suggested that varicocele may exert progressive effects on testicular function over time [4]. Our findings demonstrated that testicular stiffness progressively decreased with increasing varicocele grade, which may reflect alterations in testicular tissue characteristics associated with disease severity. A study of 69 patients who underwent microscopic varicocelectomy found that those with a larger preoperative testicular volume and lower stiffness showed a more significant improvement in semen quality after surgery [38]. This indicates that fertility outcomes may be optimized through timely surgical intervention when testicular volume is preserved and stiffness begins to decline, as the testes have not yet entered the stage of fibrosis. Fuschi et al. [14]studied 82 patients who underwent varicocele surgery and analysed their testicular SWE values, volumes, semen parameters, and pathological changes before and six months postoperatively. The postoperative SWE values decreased compared with preoperative levels, aligning with improvements in testicular histopathology. Our findings suggest that the combination of testicular volume and SWE measurements may serve as an auxiliary indicator for evaluating testicular parenchymal alterations. These parameters may provide additional information for the clinical assessment of patients with varicocele.

This study has several limitations. On the one hand, all measurements were obtained by a single operator, which reduces inter–operator variability but means that reproducibility across different operators was not evaluated. In addition, the study was conducted at a single center using a single ultrasound system, which ensures consistent and measurement conditions, but the results may not be directly generalizable to other clinical settings or ultrasound devices. Large multicenter studies using different ultrasound machines are warranted to further validate these findings. Moreover, SWE measurements were obtained only from the central portion of the testis rather than from multiple regions of the entire testicular parenchyma, potentially missing stiffness variations throughout the entire testis. In addition, the equipment used cannot automatically calculate reliability metrics such as the reliability measurement index. This may limit the objective assessment of measurement quality and lead to potential variability in stiffness measurement results. On the other hand, the sample size was insufficient, particularly in the varicocele group, and we did not include patients with right–sided or bilateral varicocele, leading to a lack of data on these groups. Furthermore, testicular biopsies were not performed, and histological confirmation of parenchymal changes was unavailable. Finally, semen parameters were not collected, limiting the ability to correlate stiffness measurements with spermatogenic function. Future studies incorporating SWE assessments and semen analysis are needed to further validate and generalize these findings. Despite these limitations, the results of this study still have potential clinical value in the assessment of patients with varicocele.

## Conclusion

In the Aixplorer system, probe frequency and ROI size have a significant effect on testicular SWE measurements. Attention should be paid when interpreting and comparing results from different measurement settings. In patients with varicocele, decreased testicular stiffness was observed despite the absence of significant changes in testicular volume. These findings suggest that SWE may provide complementary information by detecting subtle changes in testicular parenchymal in varicocele patients.

## Data Availability

The datasets generated and used in this study are available from the corresponding author on reasonable request.

## Abbreviations

VC: Varicocele
SWE: Shear wave elastography
ROI: Region of interest
Grade I, II, and III: Varicocele grades I, II, and III
CV: Coefficient of variation
CDFI: Color doppler flow imaging
SWV: Shear wave velocity
TAI: Testicular atrophy index
ARFI: Acoustic radiation force impulse

## References

[1] Wang L, Huang X, Ding Y. Shear wave elastography in assessing stiffness and volume of varicocele-affected and normal testes: a systematic review and meta-analysis. Basic Clin Androl. 2025;35(1):32. 10.1186/s12610-025-00282-1. 40859142 10.1186/s12610-025-00282-1 PMC12382270

[2] Wang K, Gao Y, Wang C, Liang M, Liao Y, Hu K. Role of oxidative stress in varicocele. Front Genet. 2022;13:850114. 10.3389/fgene.2022.850114. 35401656 10.3389/fgene.2022.850114 PMC8984266

[3] Teng W, Xiao J, Xu Q, Li P. Influence of varicocelectomy on assisted reproductive technology outcomes of infertile patients with varicocele: a systematic review and meta-analysis. Am J Mens Health. 2025;19(2):15579883251334560. 10.1177/15579883251334561. 10.1177/15579883251334561 PMC12038214 40293106

[4] Cozzolino DJ, Lipshultz LI. Varicocele as a progressive lesion: positive effect of varicocele repair. Hum Reprod Update. 2001;7(1):55–8. 10.1093/humupd/7.1.55. 11212075 10.1093/humupd/7.1.55

[5] Report on varicocele and infertility. a committee opinion. Fertil Steril. 2014;102(6):1556–60. 10.1016/j.fertnstert.2014.10.007. 25458620 10.1016/j.fertnstert.2014.10.007

[6] Leushuis E, van der Steeg JW, Steures P, Repping S, Bossuyt PM, Blankenstein MA, et al. Reproducibility and reliability of repeated semen analyses in male partners of subfertile couples. Fertil Steril. 2010;94(7):2631–5. 10.1016/j.fertnstert.2010.03.021. 20434148 10.1016/j.fertnstert.2010.03.021

[7] Lipshultz LI, Corriere JN Jr. Progressive testicular atrophy in the varicocele patient. J Urol. 1977;117(2):175–6. 10.1016/s0022-5347(17)58387-1. 833961 10.1016/s0022-5347(17)58387-1

[8] Bertolotto M, Freeman S, Richenberg J, Belfield J, Dogra V, Huang DY, et al. Ultrasound evaluation of varicoceles: systematic literature review and rationale of the ESUR-SPIWG guidelines and recommendations. J Ultrasound. 2020;23(4):487–507. 10.1007/s40477-020-00509-z. 32720266 10.1007/s40477-020-00509-z PMC7588576

[9] Saleh R, Mahfouz RZ, Agarwal A, Farouk H. Histopathologic patterns of testicular biopsies in infertile azoospermic men with varicocele. Fertil Steril. 2010;94(6):2482–5. 10.1016/j.fertnstert.2010.03.026. 20416871 10.1016/j.fertnstert.2010.03.026

[10] Gennisson JL, Deffieux T, Fink M, Tanter M. Ultrasound elastography: principles and techniques. Diagn Interv Imaging. 2013;94(5):487–95. 10.1016/j.diii.2013.01.022. 23619292 10.1016/j.diii.2013.01.022

[11] Ferraioli G, Filice C, Castera L, Choi BI, Sporea I, Wilson SR, et al. WFUMB guidelines and recommendations for clinical use of ultrasound elastography: part 3: liver. Ultrasound Med Biol. 2015;41(5):1161–79. 10.1016/j.ultrasmedbio.2015.03.007. 25800942 10.1016/j.ultrasmedbio.2015.03.007

[12] Săftoiu A, Gilja OH, Sidhu PS, Dietrich CF, Cantisani V, Amy D, et al. The EFSUMB guidelines and recommendations for the clinical practice of elastography in non-hepatic applications: update 2018. Ultraschall Med. 2019;40(4):425–53. 10.1055/a-0838-9937. 31238377 10.1055/a-0838-9937

[13] Roy C, de Marini P, Labani A, Leyendecker P, Ohana M. Shear-wave elastography of the testicle: potential role of the stiffness value in various common testicular diseases. Clin Radiol. 2020;75(7):560.e9-.e17. 10.1016/j.crad.2020.02.016. 32248949 10.1016/j.crad.2020.02.016

[14] Fuschi A, Capone L, Abuorouq S, Al Salhi Y, Velotti G, Aversa S, et al. Shear wave elastography in varicocele patients: prospective study to investigate correlation with semen parameters and histological findings. Int J Clin Pract. 2021;75(3):e13699. 10.1111/ijcp.13699. 32910514 10.1111/ijcp.13699

[15] Turna O, Aybar MD. Testicular stiffness in varicocele: evaluation with shear wave elastography. Ultrasonography. 2020;39(4):350–5. 10.14366/usg.19087. 32326674 10.14366/usg.19087 PMC7515660

[16] Dede O, Teke M, Daggulli M, Utangaç M, Baş O, Penbegül N. Elastography to assess the effect of varicoceles on testes: a prospective controlled study. Andrologia. 2016;48(3):257–61. 10.1111/and.12440. 26011193 10.1111/and.12440

[17] Bozkurt YE, Gumus BH, Ozbay M, Duzgun F, Taneli F, Kurutep S. The relationship of testicular sonoelastography with gonadotropin hormone levels and sperm parameters. Niger J Clin Pract. 2023;26(5):586–90. 10.4103/njcp.njcp_390_22. 37357474 10.4103/njcp.njcp_390_22

[18] Sakamoto H, Saito K, Oohta M, Inoue K, Ogawa Y, Yoshida H. Testicular volume measurement: comparison of ultrasonography, orchidometry, and water displacement. Urology. 2007;69(1):152–7. 10.1016/j.urology.2006.09.012. 17270639 10.1016/j.urology.2006.09.012

[19] Chiou RK, Anderson JC, Wobig RK, Rosinsky DE, Matamoros A Jr., Chen WS, et al. Color doppler ultrasound criteria to diagnose varicoceles: correlation of a new scoring system with physical examination. Urology. 1997;50(6):953–6. 10.1016/S0090-4295(97)00452-4. 9426729 10.1016/S0090-4295(97)00452-4

[20] Dubin L, Amelar RD. Varicocele size and results of varicocelectomy in selected subfertile men with varicocele. Fertil Steril. 1970;21(8):606–9. 10.1016/S0015-0282(16)37684-1. 5433164 10.1016/s0015-0282(16)37684-1

[21] Dillman JR, Chen S, Davenport MS, Zhao H, Urban MW, Song P, et al. Superficial ultrasound shear wave speed measurements in soft and hard elasticity phantoms: repeatability and reproducibility using two ultrasound systems. Pediatr Radiol. 2015;45(3):376–85. 10.1007/s00247-014-3150-6. 25249389 10.1007/s00247-014-3150-6 PMC4346477

[22] Franchi-Abella S, Corno L, Gonzales E, Antoni G, Fabre M, Ducot B, et al. Feasibility and diagnostic accuracy of supersonic shear-wave elastography for the assessment of liver stiffness and liver fibrosis in children: a pilot study of 96 patients. Radiology. 2016;278(2):554–62. 10.1148/radiol.2015142815. 26305193 10.1148/radiol.2015142815

[23] Chang S, Kim MJ, Kim J, Lee MJ. Variability of shear wave velocity using different frequencies in acoustic radiation force impulse (ARFI) elastography: a phantom and normal liver study. Ultraschall Med. 2013;34(3):260–5. 23023455 10.1055/s-0032-1313008

[24] Ferraioli G, Barr RG, Farrokh A, Radzina M, Cui XW, Dong Y, et al. How to perform shear wave elastography. Part I. Med Ultrason. 2022;24(1):95–106. 10.11152/mu-3217. 33945590 10.11152/mu-3217

[25] Rominger MB, Kälin P, Mastalerz M, Martini K, Klingmüller V, Sanabria S, et al. Influencing Factors of 2D Shear Wave Elastography of the Muscle - An Ex Vivo Animal Study. Ultrasound Int Open. 2018;4(2):E54-e60. 10.1055/a-0619-6058. 30250941 10.1055/a-0619-6058 PMC6148312

[26] Lin YY, Mao L, Li J, Zhu ZM, Luo YH, Zhou XH, et al. Exploring the anatomical factors influencing testes elasticity via ultrasound shear wave elastography: preliminary results. Rev Int Androl. 2023;21(4):100367. 10.1016/j.androl.2023.100367. 37422973 10.1016/j.androl.2023.100367

[27] Chen Q, Shi B, Zheng Y, Hu X. Analysis of influencing factors of shear wave elastography of the superficial tissue: a phantom study. Front Med. 2022;9:943844. 10.3389/fmed.2022.943844. 10.3389/fmed.2022.943844 PMC9393305 36004380

[28] Yavuz A, Yokus A, Taken K, Batur A, Ozgokce M, Arslan H. Reliability of testicular stiffness quantification using shear wave elastography in predicting male fertility: a preliminary prospective study. Med Ultrason. 2018;20(2):141–7. 10.11152/mu-1278. 29730678 10.11152/mu-1278

[29] Erdogan H, Durmaz MS, Arslan S, Gokgoz Durmaz F, Cebeci H, Ergun O, et al. Shear Wave Elastography Evaluation of Testes in Patients With Varicocele. Ultrasound Q. 2020;36(1):64–8. 10.1097/ruq.0000000000000418. 30724872 10.1097/RUQ.0000000000000418

[30] Salbas A, Büyüktoka RE. Testicular Stiffness and Volume in Varicocele Patients: A Prospective Comparative Shear Wave Elastography Study. Diagnostics (Basel, Switzerland). 2025. 10.3390/diagnostics15172150. 40941638 10.3390/diagnostics15172150 PMC12428606

[31] Li K, Liu X, Huang Y, Liu X, Song Q, Wang R. Evaluation of testicular spermatogenic function by ultrasound elastography in patients with varicocele-associated infertility. Am J Transl Res. 2021;13(8):9136–42. 34540028 PMC8430184

[32] Jedrzejewski G, Osemlak P, Wieczorek AP, Nachulewicz P. Prognostic values of shear wave elastography in adolescent boys with varicocele. J Pediatr Urol. 2019;15(3):223.e1-.e5. 10.1016/j.jpurol.2019.01.008. 30777658 10.1016/j.jpurol.2019.01.008

[33] Liu L, Xia JK, Zhu ZX, Chen WT, Xu Q. Preliminary study of virtual touch tissue imaging quantification in diffuse testicular diseases of male infertility. Acta Histochem. 2022;124(2):151860. 10.1016/j.acthis.2022.151860. 35131591 10.1016/j.acthis.2022.151860

[34] Alperen K, Ayca S, Unal T, Han GK, Sadik G. Testes parenchymal shear wave elastography findings in varicocele. J Coll Physicians Surg Pak. 2022;32(7):855–9. 10.29271/jcpsp.2022.07.855. 35795931 10.29271/jcpsp.2022.07.855

[35] Baleato-Gonzalez S, Osorio-Vazquez I, Flores-Ríos E, Santiago-Pérez MI, Laguna-Reyes JP, Garcia-Figueiras R. Testicular Evaluation Using Shear Wave Elastography (SWE) in Patients with Varicocele. Journal of imaging. 2023. 10.3390/jimaging9090166. 37754930 10.3390/jimaging9090166 PMC10532404

[36] Yüzkan S, Çilengir AH. Shear wave elastography for assessment of testicular stiffness in patients with varicocele: a prospective comparative study. J Med Ultrasound. 2022;30(4):277–81. 10.4103/jmu.jmu_218_21. 36844770 10.4103/jmu.jmu_218_21 PMC9944817

[37] Hadziselimovic F, Herzog B, Liebundgut B, Jenny P, Buser M. Testicular and vascular changes in children and adults with varicocele. J Urol. 1989;142(2 Pt 2):583–5. 10.1016/s0022-5347(17)38823-7. 2746782 10.1016/s0022-5347(17)38823-7

[38] Fu W, Cui J, Tang S. The role of testicular stiffness derived from shear wave elastography in the assessment of spermatogenesis in men with varicocele. Quant Imaging Med Surg. 2024;14(7):4987–97. 10.21037/qims-24-8. 39022243 10.21037/qims-24-8 PMC11250289

